# Supplementary material for: Flaxseed Lignans as Important Dietary Polyphenols for Cancer Prevention and Treatment: Chemistry, Pharmacokinetics, and Molecular Targets
Source: Pharmaceuticals (Basel). 2019 May 5;12(2):68. doi: 10.3390/ph12020068 (PMC6630319; doi:10.3390/ph12020068)
Supplement: Supplementary file 1 [file pharmaceuticals-12-00068-s001.zip › pharmaceuticals-479428-suppl/New Supplementary Files - Review by F.D. and J.A/Supplementary Figure 1 - Review by F.D. and J.A. (Final-2).docx]

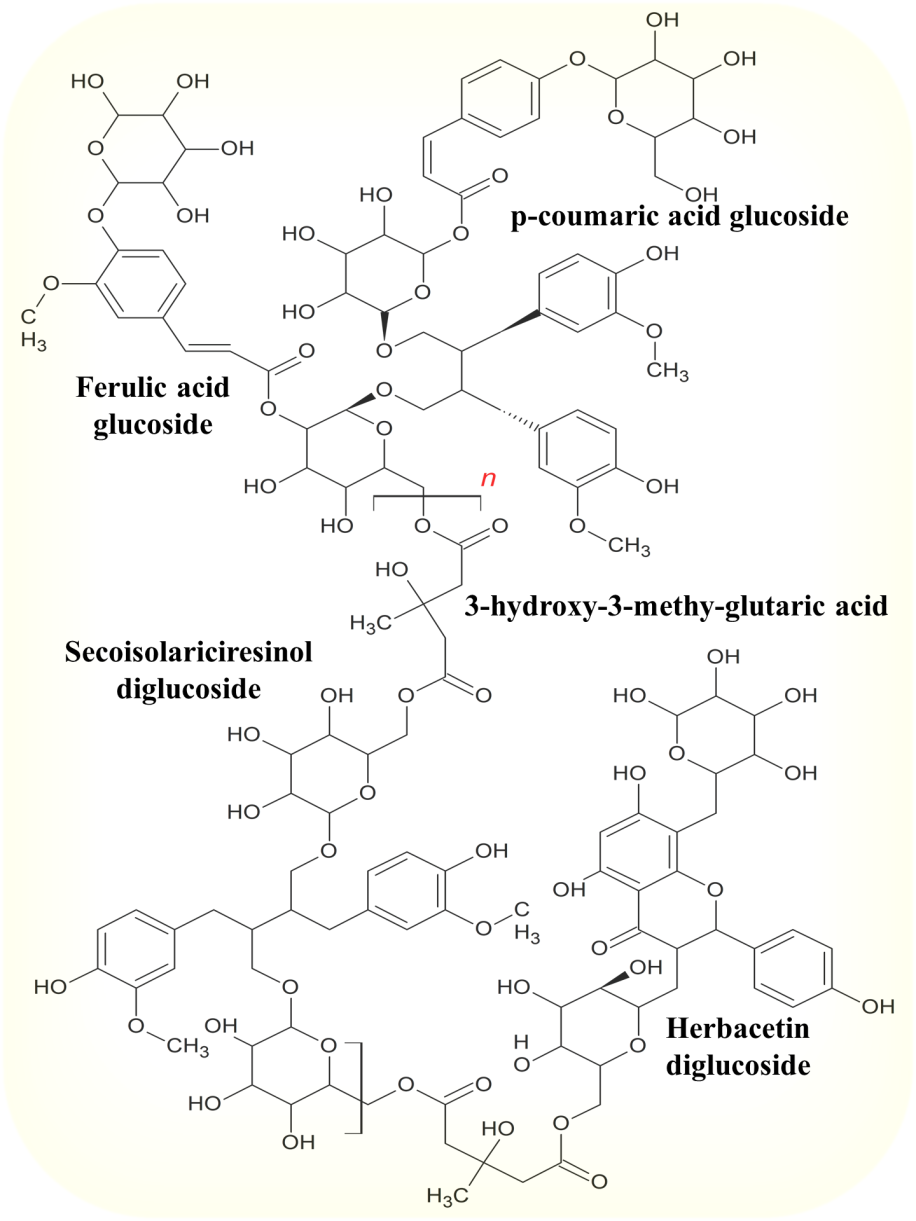


**Figure S1. Flaxseed Lignan Seocisolariciresinol Oligomer Chemical Structure.** Plant lignan secoisolariciresinol is found as a diglucoside as a part of an ester-linked complex (esterified oligomer) along with 3-hydroxyl-3-methylglutaric acid, cinnamic acid glycosides such as ferulic or p-coumaric acid as terminal units, and the flavonoid herbacetin. *Adopted from references* [1-4]

*Adopted from references:*

1. Struijs, K.; Vincken, J.P.; Doeswijk, T.G.; Voragen, A.G.; Gruppen, H. The chain length of lignan macromolecule from flaxseed hulls is determined by the incorporation of coumaric acid glucosides and ferulic acid glucosides. *Phytochemistry* **2009**, *70*, 262-269, doi:10.1016/j.phytochem.2008.12.015.

2. Struijs, K.; Vincken, J.P.; Verhoef, R.; van Oostveen-van Casteren, W.H.; Voragen, A.G.; Gruppen, H. The flavonoid herbacetin diglucoside as a constituent of the lignan macromolecule from flaxseed hulls. *Phytochemistry* **2007**, *68*, 1227-1235, doi:10.1016/j.phytochem.2006.10.022.

3. Struijs, K.; Vincken, J.P.; Verhoef, R.; Voragen, A.G.; Gruppen, H. Hydroxycinnamic acids are ester-linked directly to glucosyl moieties within the lignan macromolecule from flaxseed hulls. *Phytochemistry* **2008**, *69*, 1250-1260, doi:10.1016/j.phytochem.2007.11.010.

4. Peterson, J.; Dwyer, J.; Adlercreutz, H.; Scalbert, A.; Jacques, P.; McCullough, M.L. Dietary lignans: physiology and potential for cardiovascular disease risk reduction. *Nutrition reviews* **2010**, *68*, 571-603, doi:10.1111/j.1753-4887.2010.00319.x.
