# Supplementary material for: Flaxseed Lignans as Important Dietary Polyphenols for Cancer Prevention and Treatment: Chemistry, Pharmacokinetics, and Molecular Targets
Source: Pharmaceuticals (Basel). 2019 May 5;12(2):68. doi: 10.3390/ph12020068 (PMC6630319; doi:10.3390/ph12020068)
Supplement: Supplementary file 1 [file pharmaceuticals-12-00068-s001.zip › pharmaceuticals-479428-suppl/New Supplementary Files - Review by F.D. and J.A/Supplementary Figure 1 - Review by F.D. and J.A. (Final-2).pdf]

Flaxseed Lignans as Important Dietary Polyphenols for Cancer Prevention and Treatment: Chemistry, Pharmacokinetics, and Molecular Targets (Review) by S. Franklyn De Silva, and Jane Alcorn. Drug Discovery & Development Research Cluster, College of Pharmacy and Nutrition, 104 Clinic Place, Health Sciences Building, University of Saskatchewan, Saskatoon, Saskatchewan (SK), S7N 2Z4, Canada

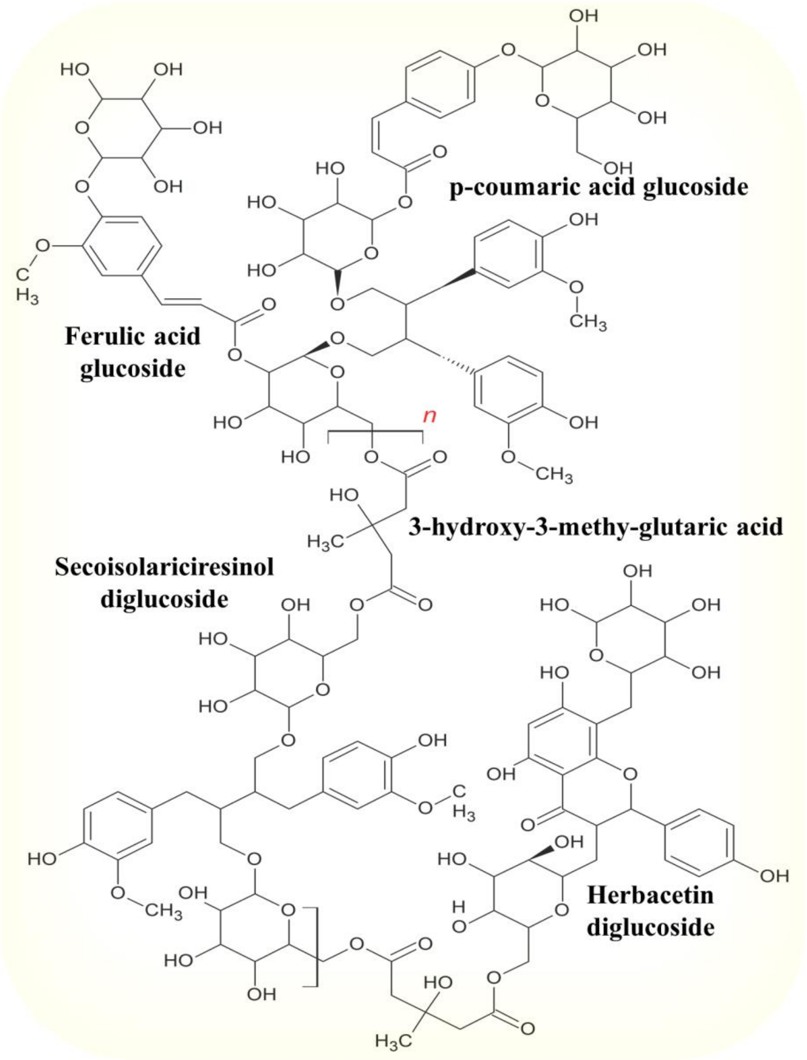

Figure S1. Flaxseed Lignan Seocisolariciresinol Oligomer Chemical Structure. Plant lignan secoisolariciresinol is found as a diglucoside as a part of an ester-linked complex (esterified oligomer) along with 3-hydroxyl-3-methylglutaric acid, cinnamic acid glycosides such as ferulic or p-coumaric acid as terminal units, and the flavonoid herbacetin. *Adopted from references* [1-4]

Adopted from references:

1. Struijs, K.; Vincken, J.P.; Doeswijk, T.G.; Voragen, A.G.; Gruppen, H. The chain length of lignan macromolecule from flaxseed hulls is determined by the incorporation of coumaric acid glucosides and ferulic acid glucosides. *Phytochemistry* **2009**, *70*, 262-269, doi:10.1016/j.phytochem.2008.12.015.
2. Struijs, K.; Vincken, J.P.; Verhoef, R.; van Oostveen-van Casteren, W.H.; Voragen, A.G.; Gruppen, H. The flavonoid herbacetin diglucoside as a constituent of the lignan macromolecule from flaxseed hulls. *Phytochemistry* **2007**, *68*, 1227-1235, doi:10.1016/j.phytochem.2006.10.022.
3. Struijs, K.; Vincken, J.P.; Verhoef, R.; Voragen, A.G.; Gruppen, H. Hydroxycinnamic acids are ester-linked directly to glucosyl moieties within the lignan macromolecule from flaxseed hulls. *Phytochemistry* **2008**, *69*, 1250-1260, doi:10.1016/j.phytochem.2007.11.010.
4. Peterson, J.; Dwyer, J.; Adlercreutz, H.; Scalbert, A.; Jacques, P.; McCullough, M.L. Dietary lignans: physiology and potential for cardiovascular disease risk reduction. *Nutrition reviews* **2010**, *68*, 571-603, doi:10.1111/j.1753-4887.2010.00319.x.
