# Supplementary material for: Flaxseed Lignans as Important Dietary Polyphenols for Cancer Prevention and Treatment: Chemistry, Pharmacokinetics, and Molecular Targets
Source: Pharmaceuticals (Basel). 2019 May 5;12(2):68. doi: 10.3390/ph12020068 (PMC6630319; doi:10.3390/ph12020068)
Supplement: Supplementary file 1 [file pharmaceuticals-12-00068-s001.zip › pharmaceuticals-479428-suppl/New Supplementary Files - Review by F.D. and J.A/Supplementary Table 1 - Review by F.D. and J.A. (Final-2).pdf]

Enterolignan/ Mammalian Lignan	Metabolic Process and Interacting Carbon (C)	Experimental Models
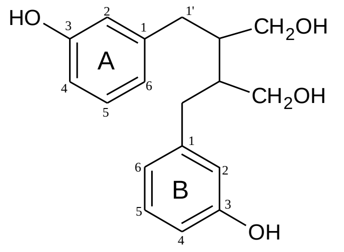 **ED** [1-6]	**Hydroxylation**<br>• 2/4/6C in ring B<br>• 2/4/5C in ring A<br>• 1′C	Human urine; Rat, pig and human liver microsomes; Rat bile and urine; Monkey hepatocytes
	**Glucuronidation** and **Sulphation**<br>• 3C in ring A	Human serum and urine
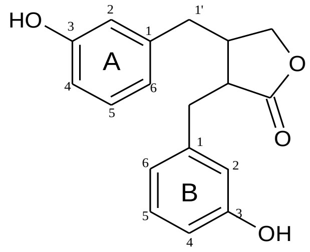 **ENL** [1-6]	**Hydroxylation**<br>• 2/5/6C in ring B<br>• 2/4/5/6C in ring A<br>• 1′C	Human urine; Rat, pig and human liver microsomes; Rat bile and urine; Monkey hepatocytes
	**Glucuronidation** and **Sulphation**<br>• 3C in ring A,B	Human serum and urine; Monkey hepatocytes

Table S1. Various Metabolites of Enterodiol (ED) and Enterolactone (ENL) Detected in Different Species : Gut microbiota mediates interconversion of plant lignans to enterolignans by varous reactions (reduction, O-deglycosylation, O-demethylation, dehydrogenation, dehydroxylation) in the gastrointestinal tract. Lignans undergo significant first pass metabolism and these molecules primarily undergo glucuronidation and sulfation in enterocytes and hepatocytes. Although the majority of metabolites are glucuronide and sulphate conjugates, a lower amount of phase I metabolites such as aromatic hydroxylated metabolites of enterodiol and enterolactone are detected in different species. This indicates that aglycone forms can be hydroxylated and further subjected to metabolism.

Adopted from references:

1. Dean, B.; Chang, S.; Doss, G.A.; King, C.; Thomas, P.E. Glucuronidation, oxidative metabolism, and bioactivation of enterolactone in rhesus monkeys. *Archives of Biochemistry and Biophysics* **2004**, *429*, 244-251, doi:https://doi.org/10.1016/j.abb.2004.06.023.
2. Niemeyer, H.B.; Honig, D.; Lange-Böhmer, A.; Jacobs, E.; Kulling, S.E.; Metzler, M. Oxidative Metabolites of the Mammalian Lignans Enterodiol and Enterolactone in Rat Bile and Urine. *Journal of Agricultural and Food Chemistry* **2000**, *48*, 2910-2919, doi:10.1021/jf0000530.
3. Jacobs, E.; Metzler, M. Oxidative metabolism of the mammalian lignans enterolactone and enterodiol by rat, pig, and human liver microsomes. *Journal of agricultural and food chemistry* **1999**, *47*, 1071, doi:10.1021/jf9809176.
4. Jacobs, E.; Kulling, S.E.; Metzler, M. Novel metabolites of the mammalian lignans enterolactone and enterodiol in human urine. *Journal of Steroid Biochemistry and Molecular Biology* **1999**, *68*, 211-218, doi:10.1016/S0960-0760(99)00033-3.
5. Knust, U.; Hull, W.E.; Spiegelhalder, B.; Bartsch, H.; Strowitzki, T.; Owen, R.W. Analysis of enterolignan glucuronides in serum and urine by HPLC-ESI-MS. *Food and Chemical Toxicology* **2006**, *44*, 1038-1049, doi:10.1016/j.fct.2005.12.008.
6. Mukker, J. Pharmacokinetic and pharmacodynamic studies on flaxseed lignans. University of Saskatchewan, Saskatoon SK Canada, 2013.
