# Supplementary material for: Flaxseed Lignans as Important Dietary Polyphenols for Cancer Prevention and Treatment: Chemistry, Pharmacokinetics, and Molecular Targets
Source: Pharmaceuticals (Basel). 2019 May 5;12(2):68. doi: 10.3390/ph12020068 (PMC6630319; doi:10.3390/ph12020068)
Supplement: Supplementary file 1 [file pharmaceuticals-12-00068-s001.zip › pharmaceuticals-479428-suppl/New Supplementary Files - Review by F.D. and J.A/Supplementary Table 2 - Review by F.D. and J.A (Final-2).docx]

**Table S2. Clinical Studies on Flaxseed/Flaxseed Lignans Administration:** Summary of sevearal relavent clinical trials pertaining to cancer prevention and treatment. Studies reported are separated into design, aim, interventions, matrix/patients, findings, and major limitations.

| **Study** | **Aim** | **Intervention** | **Matrix/patients** | **Findings** | **Major Limitations** | **Reference** |
| --- | --- | --- | --- | --- | --- | --- |
| Dietary flaxseed alters tumor biological markers in postmenopausal breast cancer | To evaluate breast cancer biomarkers when dietary flaxseed was administered to postmenopausal breast cancer patients | Flaxseed (25 g) in muffins | Postmenopausal breast cancer patients | - Reduction in breast cancer biomarkers | - Benefits are mostly attributed to lignans rather than other constituents - Attribution is based on large body of research data from animal studies | [1] |
| Dietary lignan intakes in relation to survival among women with breast cancer: the Western New York exposures and breast cancer (WEB) Study | To evaluate the association between lignan intake and breast cancer reduction after diagnosis. | Dietary lignan assessment over a period of 5 years. | Women diagnosed with breast cancer | - Reduction in mortality and morality in breast cancer diagnosed women patients | - This is an uncontrolled study and association could be an artifact - Effect of variation in lignan content due to different food consumption may have affected the outcomes of the study | [2] |
| Effect of flaxseed consumption on urinary estrogen metabolites in postmenopausal women | To evaluate the protective effect of flaxseed consumption on breast cancer in post-menopausal women | Ground flaxseed (5 and 10 g) | Postmenopausal women | - Flaxseed intake (5-10g of flaxseed /day) caused reduction in levels of 16-α hydroxyestrone (a marker for increased cancer risk) | - Reduction in biomarkers may or may not indicate the clinical protection against cancer | [3] |
| Effects of dietary flaxseed lignan extract on symptoms of benign prostatic hyperplasia (BPH) | To investigate the effect of BeneFlax on the clinical symptoms of BPH patients | BeneFlax (flaxseed lignan extract containing ~33% SDG ) at the dose equivalent to 300 and 600 mg/day of SDG | BPH patients | - Reduction in lower urinary tract syndrome (LUTS) in BPH patients | - Possibility of contribution of other BeneFlax constituents except SDG is ignored | [4] |
| Pilot study of dietary fat restriction and flaxseed supplementation in men with prostate cancer before surgery: exploring the effects on hormonal levels, prostate-specific antigen, and histopathologic features | Effect of flaxseed consumption and fat restriction in prostate cancer patients | Flaxseed (30 g/day) as a Alena Drink along with fat restricted diet | Prostate cancer patients | - Modulate several prostate cancer biomarkers such as total testosterone, free androgen index and prostate specific androgen index. | - These outcomes may be associated with fat restriction diet - Administered as drink - Study has no control group - Effect of other constituents of the drink is not accounted for. | [5] |
| Flaxseed supplementation (no dietary fat restriction) reduces prostate cancer proliferation rates in men pre-surgery | To evaluate the effects of flaxseed and fat restriction diet in prostate cancer patients | Flaxseed (10 g for 1-3 days, 20 g for 4-6 days and 30 g for 7 and beyond) | Prostate cancer patients | - lower number of Ki-67 positive cells in flaxseed treated arm | - No placebo control - Interaction between study arms were not assessed - Patient sample is biased | [6] |
| Effect of low-fat diets on plasma levels of NF-κB–regulated inflammatory cytokines and angiogenic factors in men with prostate cancer | To evaluate the changes in inflammatory biomarkers in flaxseed and low fat treated prostate cancer patients | Flaxseed (30 g/day) and low fat diet (<20% total energy) | Prostate cancer patients | - Cytokines and angiogenic factor changes in low fat diet - No changes in flaxseed treated group | - Limited number of biomarkers | [7] |
| Dietary flaxseed lignan extract lowers plasma cholesterol and glucose concentrations in hypercholestero-laemic subjects | To evaluate the effect of SDG on lipid biomarkers and glucose levels in hypercholest-erolaemic subjects | BeneFlax (flaxseed lignan extract containing ~33% SDG ) at the dose equivalent to 300 and 600 mg/day of SDG | Hypercholestero-laemic patients | - Dose dependent reduction in Total cholesterol (TC), low density lipoprotein (LDL-C) - 25% reduction in fasting glucose levels | - Study population lacks diversity - No diet restriction - Uncontrolled study | [8] |
| Flaxseed lignan lowers blood cholesterol and decreases liver disease risk factors in moderately hypercholesterole-mic men | To evaluate the effects of low dose of SDG (20 and 100 mg) in hypercholesterol-aemic patients | Flaxseed lignan capsules (equivalent to 20 and 100 mg of SDG) | Hypercholesterol-aemic patients | - Reduction in the ratio of LDL-C/HDL-C and TC | - Small population sample - Effect of other constituents of lignan capsule is negated | [9] |
| A Lignan complex isolated from flaxseed does not affect plasma lipid concentrations or antioxidant capacity in healthy postmenopausal women | To evaluate the effect of lignan extract on blood lipid levels and antioxidant capacity in Postmenopausal Women | Flaxseed lignan complex (equivalent to 500 mg/d of SDG) | Healthy Postmenopausal Women | - No change in the levels of biomarkers such as TG, TC, LDL-C and HDL-C | - Benefits may only appear in population having oxidative stress or in hypercholesterol-aemic patients | [10] |
| Effects of a flaxseed-derived lignan supplement in type 2 diabetic patients: A randomized, double-blind, cross-over trial | To evaluate the effect of flaxseed lignan complex on glucose levels, lipid profile and insulin resistance in type II diabetic patients | Flaxseed lignan supplement (360 mg/day SDG) | Type II diabetic patients | - HbA1c levels were significantly reduced - No change in lipid profile and glucose levels were observed | - Randomisation was not based on basal value of lipid profile - Changes in HbA_1c_ levels may be an artifact and may not be translated in clinical changes | [11] |
| Effects of a flaxseed-derived lignan supplement on C-reactive protein, IL-6 and retinol-binding protein 4 in type 2 diabetic patients | To evaluate different biomarkers of diabetes in diabetic patients, administering flaxseed lignan capsules | Flaxseed lignan supplement (360 mg/day SDG) | Type II diabetic patients | - Suppressed the levels of C-reactive protein in flaxseed treated group along with no changes in interleukin-6 (IL-6) and retinol binding protein-4 (RBP-4). | - Dose dependent effect was not shown | [12] |
| A randomized controlled trial of the effects of flaxseed lignan complex on metabolic syndrome composite score and bone mineral in older adults | To investigate the metabolic aspects of flaxseed lignan complex in post-menopausal women | Flaxseed lignan complex (equivalent to 543 mg/day SDG) | Healthy men and post-menopausal women | - Significant reduction in diastolic blood pressure than placebo - Decrease in composite score of metabolic syndrome in males and no change in females | - Base line diastolic blood pressure values in the placebo control and treated group are different. | [13] |

Table Primary Source: [14]

Table References:

1. Thompson, L.U.; Chen, J.M.; Li, T.; Strasser-Weippl, K.; Goss, P.E. Dietary flaxseed alters tumor biological markers in postmenopausal breast cancer. *Clin Cancer Res* **2005**, *11*, 3828-3835, doi:10.1158/1078-0432.CCR-04-2326.

2. McCann, S.E.; Thompson, L.U.; Nie, J.; Dorn, J.; Trevisan, M.; Shields, P.G.; Ambrosone, C.B.; Edge, S.B.; Li, H.F.; Kasprzak, C., et al. Dietary lignan intakes in relation to survival among women with breast cancer: the Western New York Exposures and Breast Cancer (WEB) Study. *Breast Cancer Res Treat* **2010**, *122*, 229-235, doi:10.1007/s10549-009-0681-x.

3. Haggans, C.J.; Hutchins, A.M.; Olson, B.A.; Thomas, W.; Martini, M.C.; Slavin, J.L. Effect of flaxseed consumption on urinary estrogen metabolites in postmenopausal women. *Nutr Cancer* **1999**, *33*, 188-195, doi:10.1207/s15327914nc330211.

4. Zhang, W.; Wang, X.; Liu, Y.; Tian, H.; Flickinger, B.; Empie, M.W.; Sun, S.Z. Effects of dietary flaxseed lignan extract on symptoms of benign prostatic hyperplasia. *J Med Food* **2008**, *11*, 207-214, doi:10.1089/jmf.2007.602.

5. Demark-Wahnefried, W.; Price, D.T.; Polascik, T.J.; Robertson, C.N.; Anderson, E.E.; Paulson, D.F.; Walther, P.J.; Gannon, M.; Vollmer, R.T. Pilot study of dietary fat restriction and flaxseed supplementation in men with prostate cancer before surgery: exploring the effects on hormonal levels, prostate-specific antigen, and histopathologic features. *Urology* **2001**, *58*, 47-52.

6. Demark-Wahnefried, W.; Polascik, T.J.; George, S.L.; Switzer, B.R.; Madden, J.F.; Ruffin, M.T.; Snyder, D.C.; Owzar, K.; Hars, V.; Albala, D.M. Flaxseed supplementation (not dietary fat restriction) reduces prostate cancer proliferation rates in men presurgery. *Cancer Epidemiology Biomarkers & Prevention* **2008**, *17*, 3577-3587.

7. Heymach, J.V.; Shackleford, T.J.; Tran, H.T.; Yoo, S.-Y.; Do, K.-A.; Wergin, M.; Saintigny, P.; Vollmer, R.T.; Polascik, T.J.; Snyder, D.C., et al. Effect of low-fat diets on plasma levels of NF-κB-regulated inflammatory cytokines and angiogenic factors in men with prostate cancer. *Cancer prevention research (Philadelphia, Pa.)* **2011**, *4*, 1590-1598, doi:10.1158/1940-6207.CAPR-10-0136.

8. Zhang, W.; Wang, X.; Liu, Y.; Tian, H.; Flickinger, B.; Empie, M.W.; Sun, S.Z. Dietary flaxseed lignan extract lowers plasma cholesterol and glucose concentrations in hypercholesterolaemic subjects. *The British journal of nutrition* **2008**, *99*, 1301-1309, doi:10.1017/S0007114507871649.

9. Fukumitsu, S.; Aida, K.; Shimizu, H.; Toyoda, K. Flaxseed lignan lowers blood cholesterol and decreases liver disease risk factors in moderately hypercholesterolemic men. *Nutrition research (New York, N.Y.)* **2010**, *30*, 441-446, doi:10.1016/j.nutres.2010.06.004.

10. Hallund, J.; Ravn-Haren, G.; Bugel, S.; Tholstrup, T.; Tetens, I. A lignan complex isolated from flaxseed does not affect plasma lipid concentrations or antioxidant capacity in healthy postmenopausal women. *J Nutr* **2006**, *136*.

11. Pan, A.; Sun, J.; Chen, Y.; Ye, X.; Li, H.; Yu, Z.; Wang, Y.; Gu, W.; Zhang, X.; Chen, X., et al. Effects of a Flaxseed-Derived Lignan Supplement in Type 2 Diabetic Patients: A Randomized, Double-Blind, Cross-Over Trial. *PLoS ONE* **2007**, *2*, e1148, doi:10.1371/journal.pone.0001148.

12. Pan, A.; Demark-Wahnefried, W.; Ye, X.; Yu, Z.; Li, H.; Qi, Q.; Sun, J.; Chen, Y.; Chen, X.; Liu, Y., et al. Effects of a flaxseed-derived lignan supplement on C-reactive protein, IL-6 and retinol-binding protein 4 in type 2 diabetic patients. *The British journal of nutrition* **2009**, *101*, 1145-1149, doi:10.1017/S0007114508061527.

13. Cornish, S.M.; Chilibeck, P.D.; Paus-Jennsen, L.; Biem, H.J.; Khozani, T.; Senanayake, V.; Vatanparast, H.; Little, J.P.; Whiting, S.J.; Pahwa, P. A randomized controlled trial of the effects of flaxseed lignan complex on metabolic syndrome composite score and bone mineral in older adults. *Appl Physiol Nutr Metab* **2009**, *34*, 89-98, doi:10.1139/h08-142.

14. Mukker, J. Pharmacokinetic and pharmacodynamic studies on flaxseed lignans. University of Saskatchewan, Saskatoon SK Canada, 2013.
