# Supplementary material for: Flaxseed Lignans as Important Dietary Polyphenols for Cancer Prevention and Treatment: Chemistry, Pharmacokinetics, and Molecular Targets
Source: Pharmaceuticals (Basel). 2019 May 5;12(2):68. doi: 10.3390/ph12020068 (PMC6630319; doi:10.3390/ph12020068)
Supplement: Supplementary file 1 [file pharmaceuticals-12-00068-s001.zip › pharmaceuticals-479428-suppl/New Supplementary Files - Review by F.D. and J.A/Supplementary Table 2 - Review by F.D. and J.A (Final-2).pdf]

Table S2. Clinical Studies on Flaxseed/Flaxseed Lignans Administration: Summary of sevearal relavent clinical trials pertaining to cancer prevention and treatment. Studies reported are separated into design, aim, interventions, matrix/patients, findings, and major limitations.

Study	Aim	Intervention	Matrix/patients	Findings	Major Limitations	Reference
Dietary flaxseed alters tumor biological markers in postmenopausal breast cancer	To evaluate breast cancer biomarkers when dietary flaxseed was administered to postmenopausal breast cancer patients	Flaxseed (25 g) in muffins	Postmenopausal breast cancer patients	• Reduction in breast cancer biomarkers	• Benefits are mostly attributed to lignans rather than other constituents<br>• Attribution is based on large body of research data from animal studies	[1]
Dietary lignan intakes in relation to survival among women with breast cancer: the Western New York exposures and breast cancer (WEB) Study	To evaluate the association between lignan intake and breast cancer reduction after diagnosis.	Dietary lignan assessment over a period of 5 years.	Women diagnosed with breast cancer	• Reduction in mortality and morality in breast cancer diagnosed women patients	• This is an uncontrolled study and association could be an artifact<br>• Effect of variation in lignan content due to different food consumption may have affected the outcomes of the study	[2]
Effect of flaxseed consumption on urinary estrogen metabolites in postmenopausal women	To evaluate the protective effect of flaxseed consumption on breast cancer in post-menopausal women	Ground flaxseed (5 and 10 g)	Postmenopausal women	• Flaxseed intake (5-10g of flaxseed /day) caused reduction in levels of 16-α hydroxyestrone (a marker for increased cancer risk)	• Reduction in biomarkers may or may not indicate the clinical protection against cancer	[3]

Study	Aim	Intervention	Matrix/patients	Findings	Major Limitations	Reference
Effects of dietary flaxseed lignan extract on symptoms of benign prostatic hyperplasia (BPH)	To investigate the effect of BeneFlax on the clinical symptoms of BPH patients	BeneFlax (flaxseed lignan extract containing ~33% SDG) at the dose equivalent to 300 and 600 mg/day of SDG	BPH patients	• Reduction in lower urinary tract syndrome (LUTS) in BPH patients	• Possibility of contribution of other BeneFlax constituents except SDG is ignored	[4]
Pilot study of dietary fat restriction and flaxseed supplementation in men with prostate cancer before surgery: exploring the effects on hormonal levels, prostate-specific antigen, and histopathologic features	Effect of flaxseed consumption and fat restriction in prostate cancer patients	Flaxseed (30 g/day) as a Alena Drink along with fat restricted diet	Prostate cancer patients	• Modulate several prostate cancer biomarkers such as total testosterone, free androgen index and prostate specific androgen index.	• These outcomes may be associated with fat restriction diet<br>• Administered as drink<br>• Study has no control group<br>• Effect of other constituents of the drink is not accounted for.	[5]
Flaxseed supplementation (no dietary fat restriction) reduces prostate cancer	To evaluate the effects of flaxseed and fat restriction diet in prostate	Flaxseed (10 g for 1-3 days, 20 g for 4-6 days and 30 g for 7 and beyond)	Prostate cancer patients	• lower number of Ki-67 positive cells in flaxseed treated arm	• No placebo control<br>• Interaction between study arms were not assessed<br>• Patient sample is	[6]

Study	Aim	Intervention	Matrix/patients	Findings	Major Limitations	Reference
proliferation rates in men pre-surgery	cancer patients				biased	
Effect of low-fat diets on plasma levels of NF-κB–regulated inflammatory cytokines and angiogenic factors in men with prostate cancer	To evaluate the changes in inflammatory biomarkers in flaxseed and low fat treated prostate cancer patients	Flaxseed (30 g/day) and low fat diet (<20% total energy)	Prostate cancer patients	• Cytokines and angiogenic factor changes in low fat diet<br>• No changes in flaxseed treated group	• Limited number of biomarkers	[7]
Dietary flaxseed lignan extract lowers plasma cholesterol and glucose concentrations in hypercholestero-laemic subjects	To evaluate the effect of SDG on lipid biomarkers and glucose levels in hypercholest-erolaemic subjects	BeneFlax (flaxseed lignan extract containing ~33% SDG) at the dose equivalent to 300 and 600 mg/day of SDG	Hypercholestero-laemic patients	• Dose dependent reduction in Total cholesterol (TC), low density lipoprotein (LDL-C)<br>• 25% reduction in fasting glucose levels	• Study population lacks diversity<br>• No diet restriction<br>• Uncontrolled study	[8]
Flaxseed lignan lowers blood cholesterol and decreases liver disease risk factors in moderately	To evaluate the effects of low dose of SDG (20 and 100 mg) in hypercholesterol-aemic patients	Flaxseed lignan capsules (equivalent to 20 and 100 mg of SDG)	Hypercholesterol-aemic patients	• Reduction in the ratio of LDL-C/HDL-C and TC	• Small population sample<br>• Effect of other constituents of lignan capsule is negated	[9]

Study	Aim	Intervention	Matrix/patients	Findings	Major Limitations	Reference
hypercholesterole-mic men						
A Lignan complex isolated from flaxseed does not affect plasma lipid concentrations or antioxidant capacity in healthy postmenopausal women	To evaluate the effect of lignan extract on blood lipid levels and antioxidant capacity in Postmenopausal Women	Flaxseed lignan complex (equivalent to 500 mg/d of SDG)	Healthy Postmenopausal Women	• No change in the levels of biomarkers such as TG, TC, LDL-C and HDL-C	• Benefits may only appear in population having oxidative stress or in hypercholesterol-aemic patients	[10]
Effects of a flaxseed-derived lignan supplement in type 2 diabetic patients: A randomized, double-blind, cross-over trial	To evaluate the effect of flaxseed lignan complex on glucose levels, lipid profile and insulin resistance in type II diabetic patients	Flaxseed lignan supplement (360 mg/day SDG)	Type II diabetic patients	• HbA1c levels were significantly reduced<br>• No change in lipid profile and glucose levels were observed	• Randomisation was not based on basal value of lipid profile<br>• Changes in HbA_{1c} levels may be an artifact and may not be translated in clinical changes	[11]
Effects of a flaxseed-derived lignan supplement on C-reactive protein, IL-6 and retinol-binding protein 4 in type 2 diabetic patients	To evaluate different biomarkers of diabetes in diabetic patients, administering flaxseed lignan capsules	Flaxseed lignan supplement (360 mg/day SDG)	Type II diabetic patients	• Suppressed the levels of C-reactive protein in flaxseed treated group along with no changes in interleukin-6 (IL-6) and retinol	• Dose dependent effect was not shown	[12]

Study	Aim	Intervention	Matrix/patients	Findings	Major Limitations	Reference
				binding protein-4 (RBP-4).		
A randomized controlled trial of the effects of flaxseed lignan complex on metabolic syndrome composite score and bone mineral in older adults	To investigate the metabolic aspects of flaxseed lignan complex in post-menopausal women	Flaxseed lignan complex (equivalent to 543 mg/day SDG)	Healthy men and post-menopausal women	• Significant reduction in diastolic blood pressure than placebo<br>• Decrease in composite score of metabolic syndrome in males and no change in females	• Base line diastolic blood pressure values in the placebo control and treated group are different.	[13]

Table Primary Source: [14]

Table References:

1. Thompson, L.U.; Chen, J.M.; Li, T.; Strasser-Weippl, K.; Goss, P.E. Dietary flaxseed alters tumor biological markers in postmenopausal breast cancer. *Clin Cancer Res* **2005**, *11*, 3828-3835, doi:10.1158/1078-0432.CCR-04-2326.
2. McCann, S.E.; Thompson, L.U.; Nie, J.; Dorn, J.; Trevisan, M.; Shields, P.G.; Ambrosone, C.B.; Edge, S.B.; Li, H.F.; Kasprzak, C., et al. Dietary lignan intakes in relation to survival among women with breast cancer: the Western New York Exposures and Breast Cancer (WEB) Study. *Breast Cancer Res Treat* **2010**, *122*, 229-235, doi:10.1007/s10549-009-0681-x.
3. Haggans, C.J.; Hutchins, A.M.; Olson, B.A.; Thomas, W.; Martini, M.C.; Slavin, J.L. Effect of flaxseed consumption on urinary estrogen metabolites in postmenopausal women. *Nutr Cancer* **1999**, *33*, 188-195, doi:10.1207/s15327914nc330211.
4. Zhang, W.; Wang, X.; Liu, Y.; Tian, H.; Flickinger, B.; Empie, M.W.; Sun, S.Z. Effects of dietary flaxseed lignan extract on symptoms of benign prostatic hyperplasia. *J Med Food* **2008**, *11*, 207-214, doi:10.1089/jmf.2007.602.
5. Demark-Wahnefried, W.; Price, D.T.; Polascik, T.J.; Robertson, C.N.; Anderson, E.E.; Paulson, D.F.; Walther, P.J.; Gannon, M.; Vollmer, R.T. Pilot study of dietary fat restriction and flaxseed supplementation in men with prostate cancer before surgery: exploring the effects on hormonal levels, prostate-specific antigen, and histopathologic features. *Urology* **2001**, *58*, 47-52.
6. Demark-Wahnefried, W.; Polascik, T.J.; George, S.L.; Switzer, B.R.; Madden, J.F.; Ruffin, M.T.; Snyder, D.C.; Owzar, K.; Hars, V.; Albala, D.M. Flaxseed supplementation (not dietary fat restriction) reduces prostate cancer proliferation rates in men presurgery. *Cancer Epidemiology Biomarkers & Prevention* **2008**, *17*, 3577-3587.
7. Heymach, J.V.; Shackleford, T.J.; Tran, H.T.; Yoo, S.-Y.; Do, K.-A.; Wergin, M.; Saintigny, P.; Vollmer, R.T.; Polascik, T.J.; Snyder, D.C., et al. Effect of low-fat diets on plasma levels of NF-κB-regulated inflammatory cytokines and angiogenic factors in men with prostate cancer. *Cancer prevention research (Philadelphia, Pa.)* **2011**, *4*, 1590-1598, doi:10.1158/1940-6207.CAPR-10-0136.
8. Zhang, W.; Wang, X.; Liu, Y.; Tian, H.; Flickinger, B.; Empie, M.W.; Sun, S.Z. Dietary flaxseed lignan extract lowers plasma cholesterol and glucose concentrations in hypercholesterolaemic subjects. *The British journal of nutrition* **2008**, *99*, 1301-1309, doi:10.1017/S0007114507871649.
9. Fukumitsu, S.; Aida, K.; Shimizu, H.; Toyoda, K. Flaxseed lignan lowers blood cholesterol and decreases liver disease risk factors in moderately hypercholesterolemic men. *Nutrition research (New York, N.Y.)* **2010**, *30*, 441-446, doi:10.1016/j.nutres.2010.06.004.
10. Hallund, J.; Ravn-Haren, G.; Bugel, S.; Tholstrup, T.; Tetens, I. A lignan complex isolated from flaxseed does not affect plasma lipid concentrations or antioxidant capacity in healthy postmenopausal women. *J Nutr* **2006**, *136*.
11. Pan, A.; Sun, J.; Chen, Y.; Ye, X.; Li, H.; Yu, Z.; Wang, Y.; Gu, W.; Zhang, X.; Chen, X., et al. Effects of a Flaxseed-Derived Lignan Supplement in Type 2 Diabetic Patients: A Randomized, Double-Blind, Cross-Over Trial. *PLoS ONE* **2007**, *2*, e1148, doi:10.1371/journal.pone.0001148.
12. Pan, A.; Demark-Wahnefried, W.; Ye, X.; Yu, Z.; Li, H.; Qi, Q.; Sun, J.; Chen, Y.; Chen, X.; Liu, Y., et al. Effects of a flaxseed-derived lignan supplement on C-reactive protein, IL-6 and retinol-binding protein 4 in type 2 diabetic patients. *The British journal of nutrition* **2009**, *101*, 1145-1149, doi:10.1017/S0007114508061527.
13. Cornish, S.M.; Chilibeck, P.D.; Paus-Jennsen, L.; Biem, H.J.; Khozani, T.; Senanayake, V.; Vatanparast, H.; Little, J.P.; Whiting, S.J.; Pahwa, P. A randomized controlled trial of the effects of flaxseed lignan complex on metabolic syndrome composite score and bone mineral in older adults. *Appl Physiol Nutr Metab* **2009**, *34*, 89-98, doi:10.1139/h08-142.
14. Mukker, J. Pharmacokinetic and pharmacodynamic studies on flaxseed lignans. University of Saskatchewan, Saskatoon SK Canada, 2013.
